# The Insurance Act and Poor-Law Infirmaries

**Published:** 1912-08-10

**Authors:** 


### The Insurance Act and Poor-Law Infirmaries.

### THE CHAIRMAN OF THE LAMBETH BOARD OF GUARDIANS GIVES HIS VIEWS.

Mr. Frank Briant, J.P., L.C.C., chairman of the Lambeth Board of Guardians and a member of the London Insurance Committee, which is responsible, and is making arrangements, for the administration of the medical benefits under the National Insurance Act, was interviewed by a representative of The Hospital with reference to the probable effects of the Act upon Poor-Law Infirmaries. In our issue of July 20 we published statistics showing the extended work done in Lambeth Infirmary, when Mr. Briant commented on the number of operations performed at the institution.

"The first effect of the Insurance Act on infirmaries," Mr. Briant said, "will be with regard to the treatment of tuberculosis, because the sections of the Act dealing with that disease come into operation at once. Provision has been made under the Act for the treatment of insured persons, but this will not touch in the main those already in institutions, because in all probability they have not been able to join any society, therefore it is right to assume there is going to be an enormous number of tuberculosis patients to be institutionally treated.

"The London Insurance Committee," continued Mr. Briant, "is forming temporary committees for each Metropolitan borough to carry out the duties of the Act as regards dispensary tubercular treatment, and these committees will start work almost immediately. The grant allowed by the Government under the Act will be sufficient for us to make arrangements with existing hospitals to set aside for our use a certain number of beds for tubercular patients."

"Will these patients now decrease in number?"

"At the Lambeth Infirmary during twelve months," said Mr. Briant, "351 persons were treated for tuberculosis, and a large number in addition for other forms of tubercular disease. It is obvious that the Act must affect the number of patients who in future will be treated in the infirmaries. Without having any actual data to rely upon, I think it is likely that a large proportion of persons who are now treated in the infirmaries will in the course of a year or two be insured under the Act, and, as it is provided that such treatment is not to be connected with a Poor-Law institution, the tendency will be a gradual diminution, and, in the end, an almost entire cessation of cases of this character in Poor-Law institutions."

### The Future Status of Infirmaries.

"Will the use and status of the infirmaries be altered?"

"With regard to the more general question of the use of infirmaries the figures given in the return of the Lambeth Infirmary, which has appeared in The Hospital, prove the increasing use of infirmaries for cases which formerly were treated in hospitals alone. When the work of the Insurance Committee is in full operation a large number of cases must be those requiring institutional treatment, and for which the accommodation of the hospitals will be insufficient, even if the hospitals were willing to take them. It is difficult quite to understand the position an insured person will be able to take under these circumstances. It may very naturally be said that as this is part of the medical treatment they should not be compelled, even if the Act allowed it, to enter a Poor-Law institution at all. I can only believe that the altered circumstances in recent years and the Insurance Act will lead to a complete alteration of the status of the present Poor-Law Infirmary, and that ultimately both hospitals and infirmaries will have to be under the same form of control and available for all classes of patients.

"The general idea that there is something degrading or pauperising in entering a Poor-Law Infirmary is 'simply a superstition.' As a matter of fact anyone entering an infirmary is compelled to pay out of his means towards the cost of his maintenance, and, further, his relatives are compelled to pay towards the expense incurred by the patient."

### The Question of Institutional Treatment.

"Then as to the general effect of the Act?"

"It is because, amongst other reasons, I believe the Insurance Act will eventually produce a complete change in the system and methods of institutional treatment that I welcome it gladly. Of late years there have been increased facilities for the special treatment of certain forms of disease. Infectious fevers, ophthalmia, ringworm, measles, and tubercular diseases are amongst the many for which special institutions are freely provided. This will apparently reduce the requirements in the way of accommodation in ordinary hospitals and infirmaries, but I am nevertheless convinced that a large number of persons, who are at present treated somewhat ineffectively in their own homes, owing largely to want of nursing and bad dietary, and not through any fault of the medical men concerned, will have to be persuaded in the future to enter some institution. The fact that under the Insurance Act there will be some provision for the home during illness will tend to make those who often continue at work to the danger of their health and recovery more ready to accept the institutional treatment they require. Comparatively small as are the grants under the Insurance Act, they will at any rate be sufficient to induce many to accept proper treatment, knowing at least there will be enough to keep the rent going and some balance left for the ordinary requirements of the home. In this respect I think that the Act will conduce to the general better health of the nation."

